# The Difference of Neural Networks between Bimanual Antiphase and In-Phase Upper Limb Movements: A Preliminary Functional Magnetic Resonance Imaging Study

**DOI:** 10.1155/2017/8041962

**Published:** 2017-06-20

**Authors:** Qiang Lin, Hai Li, Yu-Rong Mao, Wai-Leung Lo, Jiang-Li Zhao, Ling Chen, Yan Leng, Dong-Feng Huang, Le Li

**Affiliations:** Department of Rehabilitation Medicine, Guangdong Engineering Technology Research Center for Rehabilitation Medicine and Clinical Translation, The First Affiliated Hospital, Sun Yat-sen University, Guangzhou, China

## Abstract

Most daily movements require some degree of collaboration between the upper limbs. The neural mechanisms are bimanual-condition specific and therefore should be different between different activities. In this study, we aimed to explore intraregional activation and interregional connectivity during bimanual movement by functional magnetic resonance imaging (fMRI). Ten right-handed, normal subjects were recruited. The neural correlates of unimanual (right side) and bimanual (in-phase and antiphase) upper limb movements were investigated. Connectivity analyses were carried out using the psychophysiological interaction (PPI) model. The cerebellum was strongly activated in both unimanual and bimanual movements, and the cingulate motor area (CMA) was the most activated brain area in antiphase bimanual movement. Moreover, compared with unimanual movement, CMA activation was also observed in antiphase bimanual movement, but not in in-phase bimanual movement. In addition, we carried out the PPI model to study the differences of effective connectivity and found that the cerebellum was more connected with the CMA during antiphase bimanual movement than in-phase bimanual movement. Our findings elucidate the differences of the cerebellar-cerebral functional connectivity between antiphase and in-phase bimanual movements, which could be used to facilitate the development of a neuroscience perspective on bimanual movement control in patients with motor impairments.

## 1. Introduction

Most daily movements require certain degree of collaboration between the upper limbs [1–3]; interlimb coordination is important for performing goal-oriented daily movements [4]. Bimanual movements, which are more abundant than unimanual movements [3], are effective instruments to investigate motor dysfunctions in general and the underlining mechanisms of asymmetry and lateralization following neurodegenerative disorders and other neurological diseases [3, 5, 6].

Previous studies have focused on two patterns due to their basic model of all-bimanual movement [7]: the in-phase pattern, arising from the homologous muscle activation, and the antiphase pattern, arising from the nonhomologous muscle activation [3]. One example of such bimanual movement is the simultaneous (in-phase) or alternative (antiphase) flexion and extension elbow movement used in our study. In-phase bimanual models are more precise and stable than antiphase models at any time [4, 8, 9], while the antiphase models are increasingly destabilized at high frequencies, even resulting in a transition to the more stable models [10]. On the other hand, control of such bimanual coordination tasks is often disrupted in patients suffering from brain pathologies. Compared with bimanual in-phase movements, patients with neurological diseases, such as Parkinson's disease, can make more errors when they perform bimanual antiphase movements [11, 12]. It is commonly agreed that bilateral arm movements are associated with extra brain circuits, for example, primary motor cortex, premotor cortex (PMC), and the supplementary motor cortex (SMA) [13–16] over and above the basal ganglia [17, 18]. In addition, some studies also suggest the crucial role of the cerebellum [19] and cingulate motor area (CMA) [20–22] in mediating the coordination of limb movements. Despite all these, previous studies suggested that (1) brain activation during bimanual movement does not reflect the sum of brain activation of left and right unimanual movements [23], (2) the same brain regions and even the same neurons respond similarly during unimanual and bimanual movements based on the electrophysiological responses [24], and (3) the most significant differences of various bimanual might be more related to the degree of interregions' connectivity [25]. Therefore, it is needed to further investigate the neural connectivity of the bimanual coordination, which may help to well understand the neural mechanism and guide the precise clinical treatment.

In this study, we aimed to explore the neurological bimanual movement mechanism by directly comparing the intraregional activation and interregional connectivity between bimanual antiphase and in-phase movements using the blood-oxygen-level dependent (BOLD) functional magnetic resonance imaging (fMRI). We chose unimanual and bimanual extension-flexion elbow movements as our tasks. Although these bimanual in-phase and antiphase movements are relatively simple for normal subjects to complete, it is still the most vulnerable impairment in motor function and the basis of motor recovery training for patients with neurological disease. Based on previous research, we hypothesized that the degree of involvement in bimanual movement would contribute to changes in intraregional activation and interregional coupling and highlight the role of the cerebellar-cerebral functional connectivity during bimanual movement.

## 2. Materials and Methods

### 2.1. Subjects

Ten normal subjects (age range, 55–63 years; mean ± SD, 59.5 ± 2.5 years; 4 men and 6 women) were recruited in our study. We recruited subjects aged 55–63 years old because the age has direct implications for the performance of everyday functional activities [26, 27] and older adults tend to decrease coordination and smoothness of movement [28]. Exclusion criteria included history of stroke, heart attacks, and psychiatric diseases. All subjects were right-handed measured with the Edinburgh handedness inventory [29] and signed their informed consent forms. The study was approved by the Ethical Committee of the First Affiliated Hospital of Sun Yat-sen University.

### 2.2. Tasks

Subjects were required to perform three types of elbow extension and flexion movement tasks: (1) unimanual movement (the right elbow movement), (2) bimanual in-phase elbow movement (both elbow movements simultaneously), and (3) bimanual antiphase movement (both elbow movements alternately). To avoid distractions from auditory and vision, we used the self-paced paradigm where all the movements were executed at an interval of 2 s. Moreover, all subjects practiced before the fMRI till they can perform the tasks correctly.

### 2.3. fMRI Procedure

A 3.0T Siemens Sonata scanner was used for our study. The echoplanar imaging gradient sequence was used, and thirty-six axial slices were collected (echo time (TE) = 25 ms, repetition time (TR) = 2000 ms, field of view (FOV) = 200 × 200 cm, flip angle = 90^o^, matrix = 64 × 64, thickness = 3 mm, and gap = 0.3 mm). Moreover, 3D T1 images were obtained (echo time/TE = 2.54 ms, repetition time/TR = 1460 ms, inversion time = 900 ms, image matrix = 256 × 256, flip angle = 9°, number of slices = 192, and thickness = 1 mm) [23]. We used the block-design paradigm, where one fMRI session and three task sessions were performed randomly for one subject. Each task session contains the “rest” and “active” conditions, and each condition lasted for 20 s. In each session, the “rest” and “active” conditions were alternatively repeated six times. During the rest condition, subjects were instructed to remain motionless, while during the active condition, subjects performed one motor task in each session. The whole process of fMRI data collection for each subject was monitored by an investigator standing within the fMRI scan room. If there were any errors during performing the task or obvious head movements, the subject was required to repeat that session until he/she could perform the movement properly.

### 2.4. Data Analysis

fMRI data were analyzed with Statistical Parametric Mapping 8 software (SPM8, http://www.fil.ion.ucl.ac.uk/spm/software/spm8/). The functional data sets were reoriented into the anterior commissure-posterior commissure (AC-PC) axis planes. Afterward, these data were preprocessed by following four steps: realignment, coregistration, normalization, and smoothing. (1) Realignment was used to correct for each subject's head motions and rotations during the scanning session, where all subjects had less than 1° of rotation in each axis and less than 3 mm maximum translation in the X, Y, or Z plane. (2) Coregistration between the structural and functional data was applied to maximize the mutual information. (3) Normalization was used to spatially realign all functional volumes into MNI (Montreal Neurological Institute) space, and then all the normalized images were resliced by 3 × 3 × 3 mm^3^ voxels. (4) Smoothing was used to smooth the normalized functional series with a 6 mm full width at half-maximum Gaussian filter. Statistics were conducted on family-wise error corrected at *P* < 0.05. For the group analysis, one-sample *t*-tests were used for the identification of the the brain activity for three movement tasks, respectively (*P* < 0.0001, noncorrected, cluster size > 10). Moreover, paired *t*-tests were used for comparison of the brain activity between bimanual and unimanual movements (antiphase bimanual and unimanual movements, in-phase bimanual and unimanual movements, and antiphase and in-phase bimanual movements), respectively (*P* < 0.001, noncorrected).

### 2.5. Regions of Interest

Because we were interested in grey matter structures, bilateral volumes of the following 9 cortical and subcortical regions were extracted and used in the statistical analyses: primary motor cortex (M1), SMA, PMC, cingulate cortex area (CMA), thalamus, caudate, putamen, pallidum, and cerebellar cortex. These 9 cortical and subcortical regions of interest (ROI) were chosen, given their incontestable involvement in motor control.

### 2.6. Functional Connectivity Analysis

Psychophysiological interaction (PPI) was used to analyze the functional connectivity in this study [30, 31]. This method indicates task-specific increases in the relationship between different brain areas' activity [30]. We used PPI to detect the difference in effective connectivity while the subject performed in-phase bimanual upper limb movements versus antiphase movements.

We chose the cerebellum as a ROI for PPI analysis given the critical role of the cerebellum in motor control and because it was consistently activated in all subjects during both unimanual and bimanual conditions. The PPI analysis used a general linear model (GLM) to examine the interaction of task condition (http://www.fil.ion.ucl.ac.uk/spm/). The fMRI time course of each selected ROI was obtained by using the first eigenvariate of a 6 mm radial sphere surrounding each peak voxel. Then, the one-sample *t*-test was used to do the group analysis for bimanual tasks (anti- and in-phase bimanual movements) respectively, and the paired *t*-test was used to compare the differences between anti- and in-phase bimanual movements in the subject group (*P* < 0.05, noncorrected) [32].

## 3. Results

### 3.1. Brain Activity Analysis

#### 3.1.1. Unimanual Movements

**Figure 1:**
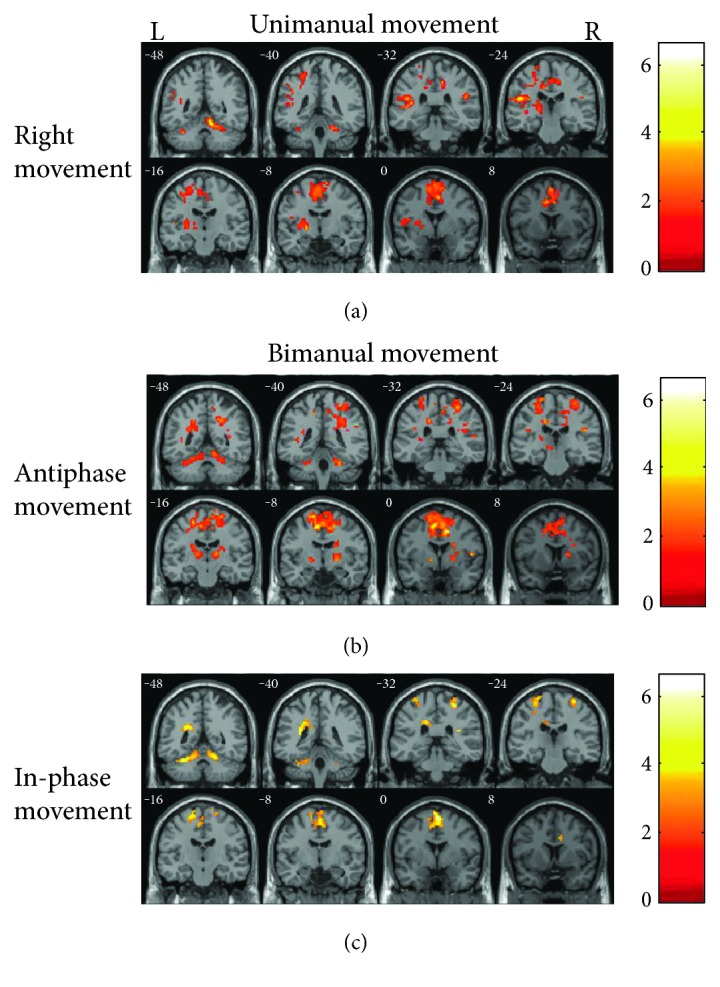
Brain regions activated during performing unimanual (right elbow) and bimanual (antiphase and in-phase) movements in the normal subject group. Results were thresholded at *p* < 0.0001, noncorrected and cluster size > 10. (a) Brain areas activated during performing unimanual movement (right elbow). (b) Brain areas activated during performing antiphase bimanual movements. (c) Brain areas activated during performing in-phase bimanual movements.

The most peak *T* values of activation were in the right cerebellum and right SMA during right-elbow movement (peak *T* values: 23.73 and 20.81 separately) (Table 1; Figure 1(a): one-sample *t*-test, *P* < 0.0001, noncorrected, cluster size > 10).

#### 3.1.2. Bimanual Movements

During the antiphase movements, normal subjects activated the right cingulum, left SMA, left PMC, bimanual cerebellum cortex, right pallidum, and bimanual thalamus, whereas during the in-phase movements, normal subjects activated the bimanual cerebellum cortex, bimanual SMA, and left PMC. Moreover, the three greatest peak *T* values of activation were in the right cingulum, left SMA, and left PMC during antiphase movement (peak *T* values: 21.56, 17.29, and 13.70, resp.) and right cerebellum, right SMA, and left cerebellum during in-phase movement (peak *T* values: 13.32, 13.01, and 12.38, resp.) (Table 1; Figures 1(b) and 1(c): one-sample *t*-test, *P* < 0.0001, noncorrected, cluster size > 10).

#### 3.1.3. Bimanual Movements versus Unimanual Movements

**Figure 2:**
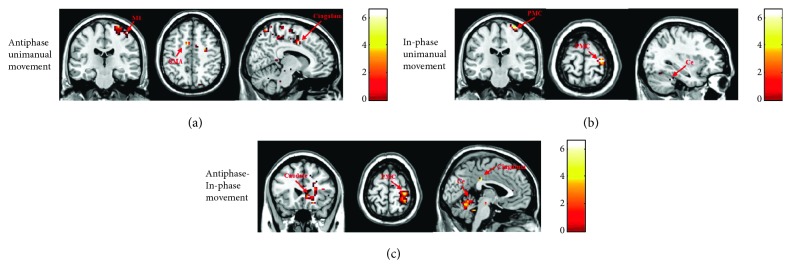
Comparison of brain regions activated during bimanual (antiphase and in-phase) and unimanual (right elbow) movements in the normal subject group. The cingulum area was strongly activated only in antiphase bimanual movements. Results were thresholded at *p* < 0.001 and noncorrected. (a) Brain areas more activated for antiphase bimanual movements than for unimanual movements in the normal subject group. (b) Brain areas more activated for in-phase bimanual movements than for unimanual movements in the normal subject group. (c) Brain areas more activated for antiphase bimanual movements than for in-phase bimanual movements in the normal subject group.

There was more activation in the bimanual cingulum, right PMC, bimanual SMA, right M1, bimanual pallidum, right putamen, right caudate, and left thalamus during antiphase bimanual movements than in unimanual movements (Table 2; Figure 2(a): paired *t*-test, *P* < 0.001, noncorrected, cluster size > 0). Additionally, there was more activation in the right PMC, left cerebellum, and right cerebellum during in-phase bimanual movements than in unimanual movements (Table 2; Figure 2(b), paired *t*-test, *P* < 0.001, noncorrected, cluster size > 0).

#### 3.1.4. Antiphase Bimanual Movements versus In-Phase Bimanual Movements

There was more activation in the right PMC and left SMA during antiphase movements than in-phase movements whereas there was more activation in the left PMC during in-phase movements than antiphase movements (Table 3; Figure 2(c): paired *t*-test, *P* < 0.001, noncorrected, cluster size > 0).

### 3.2. Functional Connectivity Analysis

PPI analysis found that, compared to in-phase movement, the cerebellum was more connected with the left cingulum, left thalamus, right thalamus, and right SMA area during antiphase movement (Table 4; paired *t*-test, *P* < 0.05, noncorrected).

## 4. Discussion

Our study was designed to evaluate the brain's intraregional activation and interregional connectivity during bimanual movements (antiphase versus in-phase). We detected that the cerebellum and SMA were strongly activated in both unimanual and bimanual movements, and the CMA was strongly activated only in antiphase bimanual movements. In addition, we utilized the PPI module to study the changes in connectivity with the cerebellum and found that the cerebellum was more connected with the CMA during antiphase movement than in-phase movement. These findings demonstrate that antiphase bimanual movement could affect the specific cerebral-cerebellar connectivity via higher CMA activation with the cerebellum.

### 4.1. Change in Brain Activity

In our study, the most brain activation was found in the CMA during bimanual antiphase movement, while in the cerebellum during unimanual movement and bimanual in-phase movement. Meanwhile, compared to unimanual movement, more highly activated CMA levels were also found in antiphase bimanual movement than in-phase bimanual movement. Our results are in agreement with a previous study from Picard and Strick, who also reported that compared to unimanual movements, bimanual movements led to a strong activation of the CMA [33]. These results indicate that the principles of interlimb coordination are unique and cannot be simply inferred from the laws of single-limb movements [5]. In other words, regions respond more strongly to bimanual movements than would be inferred from summing up the responses to the unimanual tasks [10]. Moreover, although bimanual tasks were used in the studies, some particular regions were only activated with sufficiently high degree of bimanual coordination [10, 34]. One of reasons might be that the antiphase bimanual movements are relatively less stable and accurate and requires more attention as compared to the in-phase bimanual movement [5, 35, 36]. Otherwise, generally, the activation of the CMA is also related to the higher-order aspects of motor behavior [37], such as higher task difficulty [38, 39] and cognitive control. Wenderoth et al. [34] used fMRI to investigate the neural correlates of different coordination efforts; their important finding was that the execution of spatially complex bimanual coordination, as compared to unimanual subtasks, activated the CMA. The CMA was activated when subjects performed spatially complex bimanual coordination movements as compared to unimanual movements. Furthermore, a lesion study from Stephan et al. showed that patients with a damaged CMA cannot perform nonsymmetrical bimanual movements, while symmetrical movements are not impaired, which indirectly supports our hypothesis that the CMA might be more specifically related to antiphase bimanual movement than in-phase bimanual movement.

### 4.2. Change in Effective Connectivity

Besides the intraregional activation analysis, we also used PPI model to investigate effective connectivity within symmetric/asymmetric bimanual upper-limb movements. In our study, we found a significant difference between antiphase and in-phase bimanual movements in the coupling between the cerebellum and CMA. In addition, the fMRI model we used in this study was made mainly reference to the methodology from Tao Wu's study on Parkinson's disease published in 2010 [35]. But the major difference between our study and Tao Wu's study is the different ROI chosen in PPI analysis (cerebellum versus SMA).

Regarding effective connectivity analyses, we chose the cerebellum as the ROI in PPI analysis for two main reasons: first, the cerebellum was strongly activated in both unimanual (right elbow) and bimanual (antiphase and in-phase) movements, which might be attributed to the anatomical connectivity of the cerebellar cortex with cerebral regions [40], and second, the critical role of the cerebellum in the coordination of limb movement [41, 42]. Previous experimental and clinical studies indicate that the cerebellum is essential for the organization and execution of the bimanual task, and dysfunction in this neural structure severely disrupts coordinated limb movements [19, 43]. Besides the cerebellum's cognitive, emotional, and sensory processing functions, one of the most striking properties of the cerebellum is its control in the timing of motor operations [40, 44–49]; the cerebellum is implicated in timing. Data from clinical populations suggest that patients with cerebellar pathology demonstrate a dysfunction in bimanual coordination tasks. For example, the lesion study from Bracewell and colleagues reported a 35-year-old man with obvious deficits in performing coordination simultaneous cyclic movements of the arm and leg on his ipsilesional side due to the unilateral cerebellar lesion [50]. Patients with cerebellar lesions were also reported to present timing errors in the activation of agonist and antagonist muscles during fast arm movements [51]. In-phase coordination is characterized by simultaneous timing of homologue muscle activation, while the antiphase coordination is characterized by the alternated timing of activation of homologue muscles. Generating accurate timing signals is essential for within-limb coordination (antiphase or in-phase bimanual movement); therefore, our investigation focused on the potential interaction between the cerebellum and other brain regions.

Bimanual coordination (antiphase versus in-phase) is characterized by precise spatial and temporal interactions between the limbs. Interhemispheric connections appear to be important for bimanual coordination. Chen and colleagues investigated the transcranial magnetic stimulation (TMS) effect on antiphase and in-phase bimanual movements and found that for the bimanual in-phase task, TMS could simultaneously reset the rhythmic movements of both hands, whereas TMS had difficulty in affecting antiphase bimanual movement [52]. In addition, damage to the cerebellum early in development can have long-term effects on movement and cognition due to the multiple cerebral-cerebellar circuits that are affected [43]. Those studies indicated that more complicated interhemispheric communication takes place in the relatively complex antiphase bimanual movement than in in-phase movement. One of the important findings of our study is that the cerebellum was more connected to the CMA during antiphase movements than in-phase movements. Although the studies related to the functional-anatomical relationship might be better to investigate the neuron network, only a few studies focused on the CMA and its neural network with the cerebellum structures. From the anatomical point of view, connections between the cerebellum and CMA comprising a cingulocerebellar circuit have been shown to exist by tracing studies [53, 54]; yet, related studies are still rare. However, this solid connection between the CMA and the cerebellum is the theoretical basis and direct support for our results. On the other hand, from the neural networks' point of view, the cerebellum and the CMA both play crucial roles in information process during complex cognitive and motor tasks [55]. Liu and colleagues carried out resting-state fMRI to study the differences in the functional connectivity and anatomical connectivity of the cerebellum between schizophrenic patients and normal controls and found that the bilateral cerebellum showed reduced functional connectivity to the bilateral CMA in patients, compared to controls [44]. The results revealed the potential functional connectivity between the cerebellum and the CMA, even though this connection was more related to cognitive modulation. In other words, in our study, normal subjects might use the similar strategy of increasing connectivity between the cerebellum and the CMA when performing antiphase bimanual movements than performing in-phase bimanual movements, since the antiphase movement needs more attention and more cognitive processing is involved, compared to the in-phase bimanual movement. The underlying servomechanism of this cingulocerebellar circuit to modulate the motor cooperation is still not fully understood and needs to be further studied. In addition, there might be another important theory of transcallosal inhibition attributed to our results. Both in-phase and antiphase movements require synchronization between the two sides of the upper limbs, but the antiphase movements additionally need contralateral movement suppression and the independence of the two movements [35]. There is an interhemispheric synchronization and disinhibition to control the coupled bimanual upper limb movement [4]. These studies [10, 56, 57] showed that the CMA suppresses the intrinsically favored coordination tendencies and facilitates less familiar bimanual movements; because of that, the CMA appears to play a more generic role related to cognitive control and response inhibition [34]. That could also be used to explain why the CMA was more activated in antiphase bimanual movement in our study. Furthermore, with the widespread use of TMS on clinical therapy, the studies on the TMS stimulation targets have also expanded from motor cortex to cerebellum [58]. Our study result of enhanced connectivity between CMA and cerebellum might be contributed to the underlying mechanism of the cerebellum's crucial role in time perception during motor processing.

### 4.3. Limitation

There are two important points regarding the limitations of this study. The first point is that we just studied right-handed, normal subjects of relatively small sample size and the subjects between 55 and 63 years old. Future research will be necessary to enroll left-handed subjects, subjects older than 63 years old (The epidemiologic study showed advanced age is the single most significant risk factor as 95% of stroke cases occur in people aged 45 years and above and 2/3 of stroke occur in those over the age of 65 years [59, 60].) and patients with upper limb dysfunction to explore the neurophysiology mechanism effects found in the present study. Secondly, considering this study was a pilot study focusing on cingulate cortex to compare the antiphase/in-phase bimanual tasks with dominant unimanual tasks, we just enrolled ten normal subjects.

## 5. Conclusion

Our study revealed that the CMA is assumed to be more involved in antiphase bimanual movement than in-phase bimanual movement by increasing the activation and effective connectivity with the cerebellum. Our findings elucidated differences in activity and connectivity due to the patterns of bimanual upper limb movements, which could be used to facilitate the development of a neuroscience perspective on bimanual movement control in patients with motor impairments.

## Figures and Tables

**Table 1:** Brain regions activated during performing unimanual movements (right elbow) and bimanual movements (antiphase and in-phase) in the normal subject group. Results were thresholded at *P* < 0.0001, noncorrected, and cluster size > 10.

Brain region	Coordinates			Peak level	
	*x*	*y*	*z*	*T* value	*Z* value
Unimanual movement					
Right cerebelum_superior	9	−52	−13	23.73	6.00
Right supplementary motor area	3	5	56	20.81	5.81
Right cerebelum_superior	27	−43	−25	17.10	5.51
Left supplementary motor area	0	2	68	16.09	5.42
Left cerebelum_superior	−33	−61	−25	14.31	5.23
Left cingulate gyrus	−6	11	41	14.18	5.22
Left pallidum	−21	−7	−1	14.11	5.21
Left thalamus	−15	−22	2	10.71	4.75
Left putamen	−30	−7	5	9.26	4.50
Antiphase movement					
Right cingulate gyrus	12	−1	44	21.56	5.86
Left supplementary motor area	−9	−4	50	17.29	5.53
Left precentral gyrus, PMC	−21	−13	62	13.70	5.16
Right cerebelum_superior	27	−40	−28	11.07	4.81
Right pallidum	21	−4	−1	10.66	4.74
Right thalamus	15	−16	8	10.60	4.73
Left thalamus	−18	−10	−1	10.05	4.64
Left cerebelum_superior	−9	−58	−13	9.67	4.58
In-phase movement					
Left cerebelum_superior	−30	−46	−25	13.32	5.11
Right supplementary motor area	3	−4	62	13.01	5.08
Left cerebelum_superior	−9	−55	−13	12.38	4.99
Left paracentral_lobule, PMC	−18	−13	65	11.46	4.87
Left supplementary motor area	−6	−19	50	11.19	4.83
Right cerebelum_superior	21	−46	−22	9.64	4.57

**Table 2:** Brain areas more activated in performing bimanual movements (antiphase or in-phase) than in performing unimanual movements in the normal subject group separately. Results were thresholded at *P* < 0.001 and noncorrected.

Brain region	Coordinates			Peak level	
	*x*	*y*	*z*	*T* value	*Z* value
Antiphase versus unimanual movement					
Right cingulate gyrus	9	2	41	7.57	4.14
Right precentral gyrus, PMC	27	−22	74	7.38	4.10
Left supplementary motor area	−9	5	47	6.33	3.81
Right precentral gyrus, M1	42	−22	62	6.30	3.81
Left pallidum	−12	−1	−1	5.58	3.58
Right pallidum	21	−4	−1	5.51	3.56
Right putamen	30	14	−1	5.24	3.46
Right caudate	21	20	14	4.69	3.26
Left cingulate gyrus	−9	−7	50	4.65	3.24
Right supplementary motor area	3	−1	53	4.54	3.20
Left thalamus	−15	−7	−1	4.34	3.11
Unimanual movement versus antiphase					
None activation					
In-phase versus unimanual movement					
Right precentral gyrus, PMC	30	−22	71	7.51	4.13
Left cerebelum_superior	−33	−40	−31	6.26	3.80
Right cerebelum_superior	12	−46	−4	4.89	3.33
Unimanual movement versus in-phase					
None activation					

**Table 3:** Brain areas more activated in performing antiphase bimanual movements than in performing in-phase bimanual movements in the normal subject group. Results were thresholded at *P* < 0.001 and noncorrected.

Brain region	Coordinates			Peak level	
	*x*	*y*	*z*	*T* value	*Z* value
Antiphase versus in-phase					
Right frontal_sup_2, PMC	24	−7	62	4.61	3.22
Left supplementary motor area	−9	−7	68	4.34	3.11
In-phase versus antiphase					
Left precentral gyrus, PMC	−30	−25	71	4.60	3.22

**Table 4:** Differences of effective connectivity in the cerebellum in the normal subject group during performing antiphase bimanual movements than in performing in-phase bimanual movements. Results were thresholded at *P* < 0.05 and noncorrected.

Brain region	Coordinates			Peak level	
	*x*	*y*	*z*	*T* value	*Z* value
Antiphase versus in-phase					
Left cerebelum_superior	−31	−40	−29	18.35	4.05
Left cingulate gyrus	−13	26	25	14.03	3.79
Left thalamus	−19	−19	16	12.18	3.65
Left cingulate gyrus	−7	−31	40	11.03	3.55
Right cerebelum_superior	11	−46	−2	10.70	3.52
Right thalamus	17	−25	13	10.64	3.51
Right supplementary motor area	2	−19	55	7.19	3.09
In-phase versus antiphase					
None activation					
